# The Extended Lifespan of *Caenorhabditis elegans rsks-1* Mutants Depends on the Peroxisomal Fatty Acid β-Oxidation Enzyme ACOX-1.2

**DOI:** 10.3390/cimb48090909

**Published:** 2026-09-04

**Authors:** Qiu Zhao, Rui Zhu, Hong-Xu Guo, Zi-Xi Li, Xin-Qian Gong, Jia-Hong Duan, Yi-Cheng Ma, Cheng-Gang Zou, Nan Wu

**Affiliations:** 1 State Key Laboratory for Conservation and Utilization of Bio-Resources in Yunnan, Key Laboratory for Southwest Microbial Diversity of the Ministry of Education, Yunnan Key Laboratory of Basic Research and Innovative Application for Green Biological Production, School of Life Science, Yunnan University, Kunming 650091, China; 13769239249@163.com (Q.Z.); zhuzhurui2021@163.com (R.Z.); gong1715063217@163.com (X.-Q.G.); jia_9608@163.com (J.-H.D.); mayc@ynu.edu.cn (Y.-C.M.); 2 Yunnan Key Laboratory of Stem Cell and Regenerative Medicine, School of Rehabilitation, Kunming Medical University, Kunming 650500, China; hxanda@126.com; 3 Department of Bioscience and Bioinformatics, School of Science, Xi’an Jiaotong-Liverpool University, Suzhou 215123, China; zixi.li22@student.xjtlu.edu.cn; 4 Yunnan Characteristic Plant Extraction Laboratory, Kunming 650106, China

**Keywords:** *Caenorhabditis elegans*, lifespan, reactive oxygen species, peroxisomal fatty acid β-oxidation

## Abstract

The peroxisome has been implicated in the processes of aging and longevity regulation. Nevertheless, the mechanism by which peroxisomes regulate longevity remains far from fully elucidated. Here, we compared reactive oxygen species (ROS) levels among five long-lived *Caenorhabditis elegans* mutants using cross-validation with four different ROS probes and observed an increase in hydrogen peroxide (H_2_O_2_) levels in *rsks-1(ok1255)* mutants. Transcriptomic analysis revealed that the genes involved in peroxisomal fatty acid β-oxidation were upregulated in the *rsks-1(ok1255)* mutants. The imbalance of H_2_O_2_ metabolism in the *rsks-1(ok1255)* mutants was found to be regulated by the peroxisomal fatty acid β-oxidation enzyme ACOX-1.2. Furthermore, lifespan assays and genetic analysis of transgenic strains demonstrated that the increases in autophagy and lifespan observed in long-lived *rsks-1(ok1255)* mutants were suppressed by *acox-1.2* RNAi. These findings uncover a previously unknown role of peroxisomal fatty acid β-oxidation in the regulation of aging and longevity.

## 1. Introduction

Peroxisomes are metabolic hubs involved in ROS scavenging, phosphatidylglycerol and sterol precursor synthesis, as well as amino acid and purine metabolism [1]. They are integral to preserving cellular homeostasis and redox balance [2]. Beyond their canonical roles in lipid catabolism and ROS metabolism, peroxisomes also function as signaling hubs. PEX2 senses ROS generated by peroxisomal β-oxidation and modulates adipose triglyceride lipase (ATGL) abundance via ubiquitination, establishing a feedback loop that couples fatty acid oxidation to lipolysis [3]. Perturbations in peroxisome biogenesis or function underlie a spectrum of severe genetic disorders, including Zellweger syndrome (ZS), neonatal adrenoleukodystrophy (NALD), infantile Refsum disease (IRD), and rhizomelic chondrodysplasia punctata (RCDP) [4]. Age-dependent decline in peroxisomal proteins (e.g., PRX-3, PRX-5, ACOX-1) correlates with aging in *Caenorhabditis elegans* (*C. elegans*) [5]. Genetic evidence underscores their functional conservation: pexgene manipulation modulates lifespan across species—knockdown of *pex1/prx-1* or *pex13/prx-13* prolongs life in flies [6], while *pex5/prx-5* deletion shortens it in yeast and negates the longevity phenotype of *drp-1*; *fzo-1* mitochondrial mutants in worms [7,8]. We previously showed that α-ketobutyrate(α-KB) prolongs lifespan in *C. elegans* via enhanced peroxisomal biogenesis and function [9]. However, the mechanistic underpinnings of peroxisomal involvement in aging remain poorly understood.

ROS refers to a class of highly reactive oxygen-containing molecules widely present in biological systems and nature, with typical examples being superoxide anion (O_2_^−^), hydrogen peroxide (H_2_O_2_), and hydroxyl radical (OH•). Supraphysiological ROS levels are cytotoxic to DNA, proteins, and lipids, triggering oxidative damage and cellular dysfunction [10]. Accumulating evidence links ROS to the execution of varied cell death programs, encompassing apoptosis, autophagy, and ferroptosis [11,12]. ROS are not merely destructive; they also function as signaling mediators in key cellular processes [10,11,13]. In *C. elegans*, this duality is evident in respiration-defective mutants *clk-1(qm30)*, *isp-1(qm150)*, and *nuo-6(qm200)*—all of which exhibit elevated ROS levels [14,15]. These mutants extend lifespan through the activity of the hypoxia-inducible factor HIF-1 [14,15]. Conversely, modest ROS elevations—induced by 2-deoxyglucose or low-dose paraquat—extend lifespan in *C. elegans* [16,17,18]. Yet, for decades, the dominant paradigm explaining aging has centered on the cumulative burden of oxidative damage [19]. These findings challenge the classical oxidative stress theory of aging, reframing ROS not as mere toxic byproducts but as tightly regulated signaling molecules. Indeed, subtle fluctuations in ROS levels and redox potential appear to drive key longevity pathways, suggesting that physiological control—rather than sheer abundance—governs their pro-longevity effects.

Very-long-chain fatty acids (VLCFA) are first shortened through β-oxidation in peroxisomes; subsequently, these shortened fatty acids are metabolized through mitochondrial β-oxidation [1,20]. Although both mitochondria and peroxisomes play an important role in fatty acid β-oxidation, there is a clear bias in the research community towards research focusing on the former rather than the latter. Peroxisomes have been demonstrated to participate in the process of β-oxidation of branched and VLCFAs, which results in the production of H_2_O_2_ by acyl-CoA oxidases (ACOXs) [10,21,22]. Peroxisomes contain enzymes such as catalases (CTLs) and glutathione peroxidases (GPXs), which can reduce H_2_O_2_ to O_2_ and H_2_O [23]. Nevertheless, in certain instances, the metabolic balance of H_2_O_2_ in peroxisomes may be disrupted. For example, the production of H_2_O_2_ via peroxisomal acyl-CoA oxidase ACOX-1.2 was found to be elevated in the long-lived *glp-1(e2141ts)* mutant worms [9]. Therefore, the role of peroxisomal fatty acid β-oxidation in the regulation of aging remains incompletely understood.

It is evident that alterations in insulin/insulin-like growth factor (IGF-1) signaling (IIS), the target of rapamycin (TOR) pathway, signals from the reproductive system, electron transport chain (ETC), and dietary restriction (DR) have been shown to significantly affect lifespan in worms [24,25,26]. Consistent with previous studies [9,14,15], the ROS levels were significantly increased in these long-lived mutant worms. Importantly, based on the transcriptomic data of long-lived mutant worms, we found that the mRNA levels of peroxisomal acyl-CoA oxidase genes were upregulated in *rsks-1 (ok1255)* and *glp-1(e2141ts)* mutant worms [9]. In this study, screening these *acox* genes revealed that *acox-1.2* RNAi significantly reduced H_2_O_2_ levels in long-lived *rsks-1(ok1255)* mutants and that both autophagy induction and lifespan extension in these worms depended on ACOX-1.2. These results indicate that H_2_O_2_ produced by peroxisomal fatty acid β-oxidation serves as a key signaling molecule in regulating longevity in *C. elegans*.

## 2. Materials and Methods

### 2.1. Strains

Bristol strain N2 was used as the standard wild-type strain. Strains used in this study included *glp-1(e2141ts)*, *eat-2(ad1116)*, *daf-2(e1370)*, *isp-1(qm150)*, *rsks-1(ok1255)*, *DA2123 (adIs2122[lgg-1p::GFP::lgg-1+rol-6(su1006)])*, and *CL2166 (dvIs19 [(pAF15) gst-4p::GFP::NLS])* were obtained from Caenorhabditis Genetics Center (CGC; http://www.cbs.umn.edu/CGC), funded by NIH Office of Research Infrastructure Programs (P40OD010440). The nematode strains *prx-11p::prx-11::gfp* and *acox-1.2p::gfp* were kindly provided by Dr. Huanhu Zhu (ShanghaiTech University). Strains were maintained on nematode growth medium (NGM) agar plates spotted with *Escherichia coli* OP50 [27]. All strains were grown at 20 °C unless specified.

### 2.2. RNA Interference for Worms

RNAi clones targeting specific genes were sourced from the Ahringer library. *E. coli* HT115(DE3) strains were grown overnight in LB supplemented with 100 µg/mL ampicillin at 37 °C, then seeded onto NGM plates containing the same antibiotic and 5 mM IPTG. Following a second overnight incubation at 25 °C to induce dsRNA expression, synchronized L1 larvae were transferred to these plates and cultured at 20 °C until reaching the L4 stage.

### 2.3. Lifespan Analysis

Lifespan assays were conducted at 20 °C using synchronized young adult worms cultured on OP50-seeded NGM plates. During the reproductive period, animals were transferred daily; thereafter, transfers occurred every three days. Survival was scored daily, with death defined as the cessation of pharyngeal pumping and lack of response to tactile stimulation. Each assay comprised three replicate plates (40–60 worms/plate), and all experiments were repeated independently three times (Table S1).

### 2.4. Age-Related Phenotypic Marker Assays in Worms

Synchronized all nematodes and transferred them to NGM agar plates seeded with *E. coli* OP50 upon reaching young adulthood; worms were maintained on these plates until adult day 10, when we quantified two age-dependent functional readouts. For pharyngeal pumping assays, we counted terminal bulb contractions of the pharynx over 30 s windows. Body bending frequency was similarly scored by tallying full body flexions within 30 s. Each measurement was performed with 30 individual worms, across three separate biological replicates [28,29].

### 2.5. Detection of ROS in Worms

The detection of ROS in worms was performed according to the previously described method [30]. Briefly, the synchronized worms were cultured to the young adult stage. Worms were incubated in Petri dishes filled with M9 buffer containing dihydroethidium (YEASEN, Shanghai, China, 10 μM), 2′,7′-dichlorofluorescein diacetate (MedChemExpress, Monmouth Junction, NJ, USA, 20 μM), MitoTracker red CM-H2XRos (YEASEN, 20 μM), or 10-acetyl-3,7-dihydroxy-phenoxazine (YEASEN, 20 μM) at 20 °C for 3 h, respectively. Then, worms were washed three times with M9 buffer and mounted onto microscope slides in M9 buffer. Fluorescence images were captured using a Zeiss Axioskop (Zeiss, Oberkochen, Germany). At least 30 worms were examined per assay in three independent experiments.

### 2.6. Autophagy Analysis

Transgenic worms carrying GFP::LGG-1 were grown on NGM agar plates seeded with E. coil OP50 in the presence. After 48 h of growth, the worms were immediately mounted onto microscope slides in M9 buffer. The slides were viewed using a Zeiss Axioskop 2 Plus (Zeiss, Oberkochen, Germany) fluorescence microscope. GFP::LGG-1 positive puncta were counted in the seam cells or the intestinal cells. At least 30 worms were examined per assay in three independent experiments.

### 2.7. Quantitative RT-PCR

Total RNA was extracted from worms with TRIzol Reagent (Invitrogen, Carlsbad, CA, USA). Random-primed cDNAs were generated by reverse transcription of the total RNA samples with SuperScript II (Invitrogen). Quantitative real-time PCR analysis was performed using SYBR Premix (Takara, Dalian, China) on a Roche LightCycler 480 System (Roche Applied Science, Penzberg, Germany). The gene *act-1* was used as the internal reference; quantification was computed by the 2^–ΔΔCt^ method after being normalized to the target gene. The primers are summarized in Table S2.

### 2.8. The Quantification of Peroxisome Numbers and Size

To assess peroxisome function and biogenesis, we employed the transgenic strain expressing *prx-11p::prx-11::gfp*. Synchronized worms were washed three times in M9 buffer, then mounted on microscope slides in a drop of the same buffer. *PRX-11::GFP* fluorescence was imaged using a Leica TCS SP8 STED confocal microscope (Leica Microsystems, Wetzlar, Germany). Z-stack projections focused on the intestinal Int1 and Int2 cells (the first two anterior rings) were used for analysis. Peroxisomes, identified as puncta ranging from 0.1 to 1.2 µm in diameter, were quantified for number and size across at least 30 worms per assay, with three independent biological replicates.

### 2.9. DAVID Enrichment Analysis

To identify enriched Gene Ontology (GO) terms and Kyoto Encyclopedia of Genes and Genomes (KEGG) pathways of the overlapping genes derived from the expression profiles of these two datasets, we used the Database for Annotation, Visualization, and Integrated Discovery (DAVID) version 6.7 (https://davidbioinformatics.nih.gov/ accessed on 10 March 2026) 77. GO terms or KEGG pathways with an adjusted *p* value < 0.01 were defined as significantly enriched.

### 2.10. RNA-Seq Analysis

To We obtained previously published transcriptomic datasets of long-lived mutant strains from the GEO database, including GSE272718, GSE93724, GSE293218, and GSE63075. The datasets were uniformly processed and subjected to differential expression analysis using the online platform OmicSmart. Differentially expressed genes were identified using the following criteria: |log_2_FC| ≥ 0.75 and *p* < 0.05.

### 2.11. Single-Nucleus RNA Sequencing (snRNA-Seq) Analysis

We followed the analytical procedures available on the online website (http://mengwanglab.org/atlas accessed on 12 June 2026). Raw FASTQ sequencing files, expression matrix, Seurat objects, and other analysis outputs can be downloaded from the Gene Expression Omnibus under accession number GSE229022.

### 2.12. Statistical Analysis

Differences in survival rates were analyzed using the log-rank test. Differences in ROS levels, age-related phenotypic marker assays, and the number of GFP::LGG-1 positive puncta were presented as mean ± standard deviation (SD), and graphs were generated using GraphPad Prism 8.0 software (GraphPad Software Inc., San Diego, CA, USA). Statistical analysis was performed using one-way analysis of variance (ANOVA) followed by a Student–Newman–Keuls test or Student’s *t* test.

## 3. Results

### 3.1. The ROS Levels Are Increased in the Long-Lived Mutant Worms

**Figure 1:**
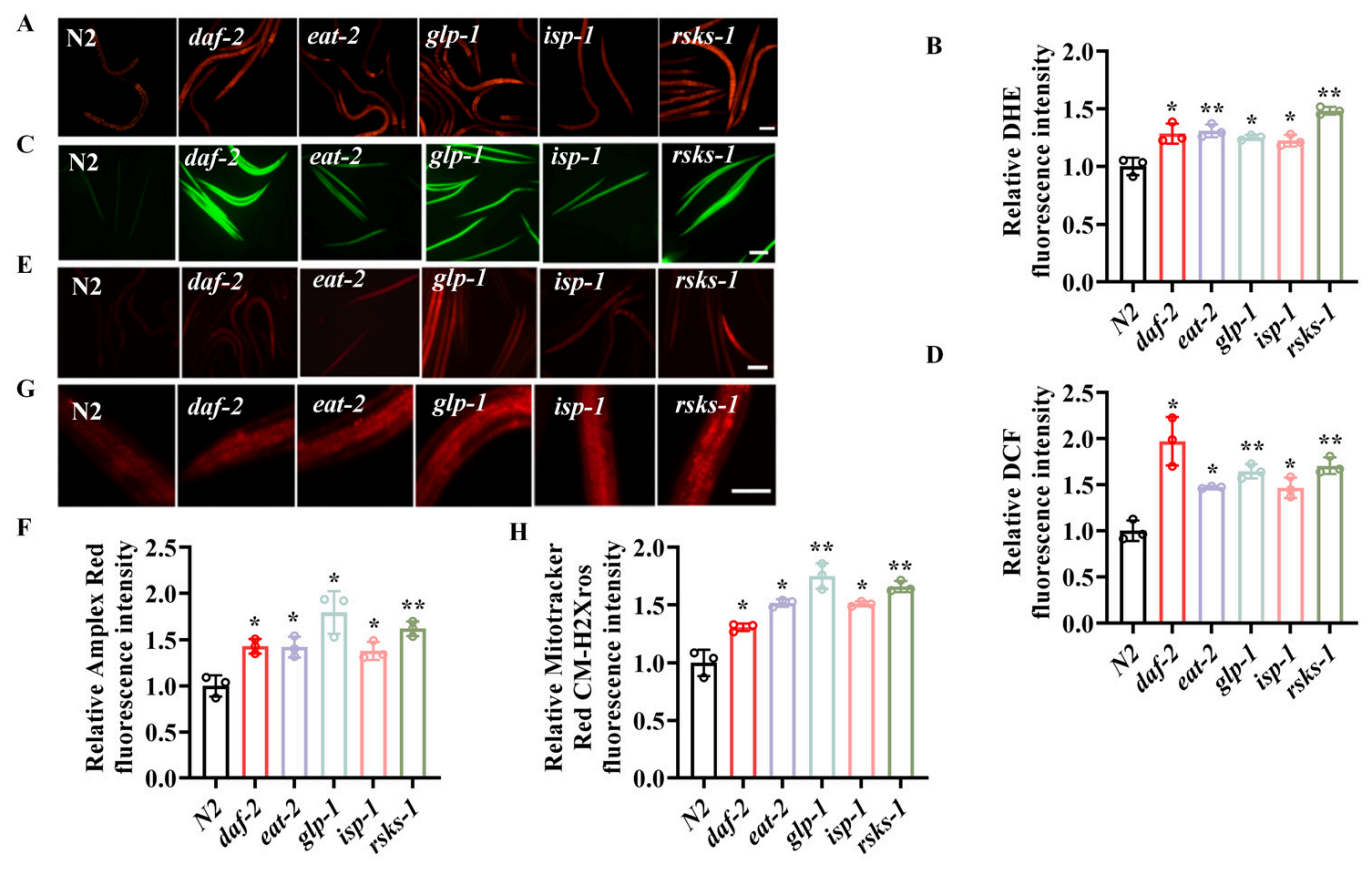
ROS metabolic homeostasis is disrupted in long-lived mutants. (**A**–**H**) The ROS levels are increased in the long-lived mutants. The long-lived mutants included *daf-2(e1370)*, *eat-2(ad1116)*, *glp-1(e2141ts)*, *isp-1(qm150)*, and *rsks-1(ok1255)*. (**A**) Representative images of dihydroethidium (DHE) staining. Scale bars: 100 μm (applies to all panels). (**B**) Quantification of the fluorescence intensity of DHE. (**C**) Representative images of 2′,7′-dichlorofluorescein diacetate (H2DCFDA) staining. Scale bars: 100 μm (applies to all panels). (**D**) Quantification of the fluorescence intensity of H2DCFDA. (**E**) Representative images of 10-acetyl-3,7-dihydroxy-phenoxazine (Amplex Red) staining. Scale bars: 100 μm (applies to all panels). (**F**) Quantification of the fluorescence intensity of Amplex Red. (**G**)Representative images of MitoTracker red CM-H2XRos staining. Scale bars: 100 μm (applies to all panels). (**H**) Quantification of the fluorescence intensity of MitoSOX Red. Data were presented as mean values ± SD of three independent experiments (*n* = 30 worms per experiment). * *p* < 0.05, ** *p* < 0.01. *p*-values are calculated using a one-way ANOVA followed by a Student’s *t* test.

To further identify the metabolic balance of ROS in long-lived mutant worms, we used 2′,7′-dichlorofluorescein diacetate (H2DCFDA) to detect oxidizing radicals and other ROS, dihydroethidium (DHE) to detect superoxide, 10-acetyl-3,7-dihydroxy-phenoxazine (Amplex Red) to detect hydrogen peroxide, and MitoTracker red CM-H2XRos to detect mitochondrial superoxide [31]. The long-lived mutants examined in this study included the *daf-2(e1370)* insulin/IGF-1 mutant [32], the *eat-2(ad1116)* nicotinic acetylcholine receptor subunit mutant [33], the *glp-1(e2141ts)* Notch receptor mutant [34], the *isp-1(qm150)* Rieske iron-sulfur protein subunit of cytochrome c oxidoreductase (complex III of the ETC) mutant [26], and the *rsks-1(ok1255)* ribosomal S6 kinase (S6K) mutant [35]. We found that the signal intensity of H2DCFDA, DHE, and Amplex Red was significantly increased in the five long-lived mutants compared to wild-type (WT) worms (Figure 1A–F). In addition, MitoSOX Red revealed that the levels of mitochondrial superoxide were elevated in the five long-lived mutants compared to WT worms (Figure 1G,H). These results suggest that ROS metabolic homeostasis is disrupted in long-lived mutants.

### 3.2. Acyl-CoA Oxidases ACOX-1.2 Is Required for H_2_O_2_ Production in rsks-1 Mutants

**Figure 2:**
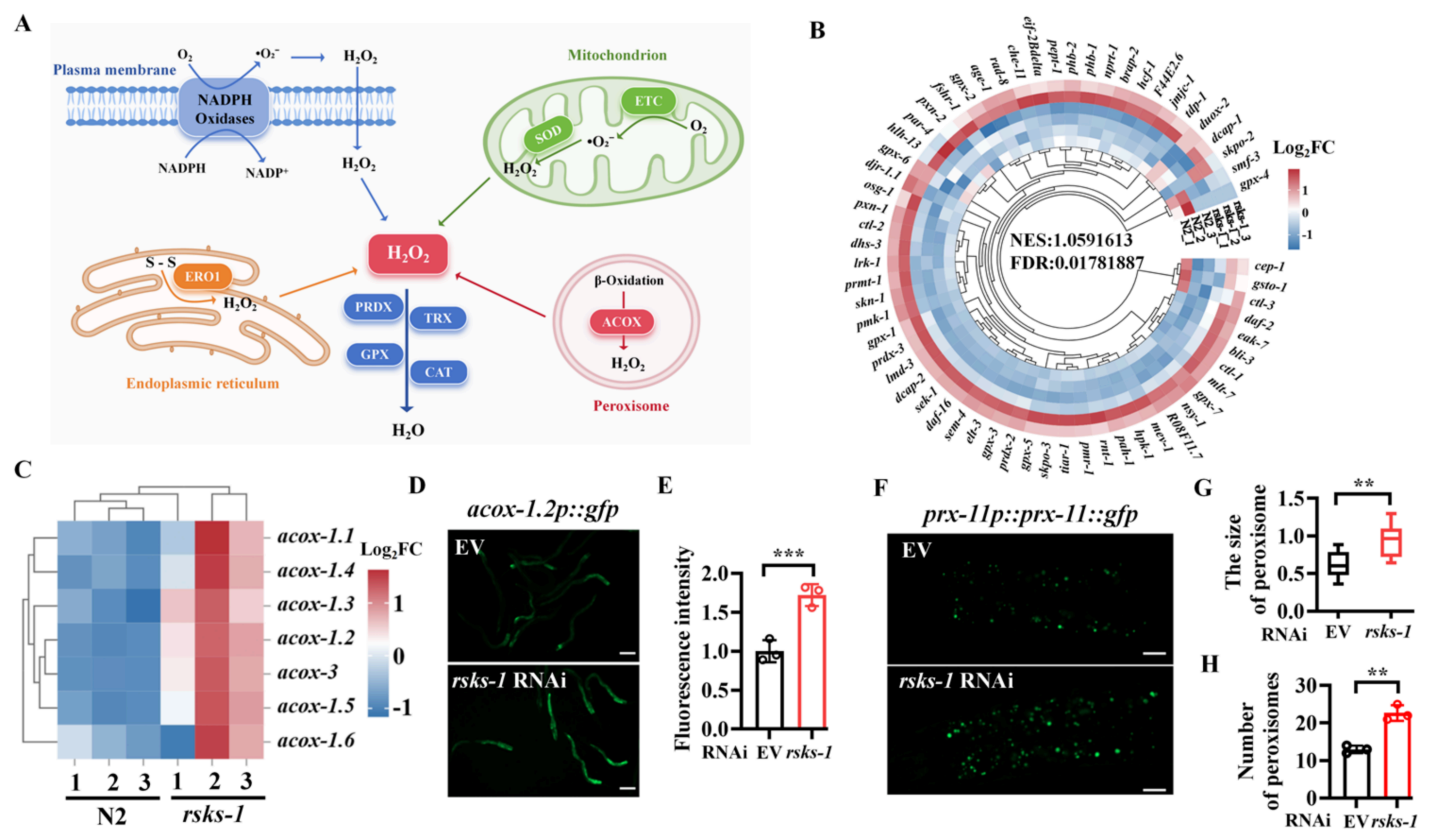
The expression of the peroxisomal acyl-CoA oxidase genes is increased in *rsks-1 (ok1255)* mutant worms. (**A**) Schematic diagram of the major sources and detoxification pathways of H_2_O_2_ in worms. (**B**) The expression pattern of the oxidative stress response genes in *rsks-1(ok1255)* mutants (GSE272718) by Gene Set Enrichment Analysis (GSEA). (**C**) The expression pattern of the acyl-CoA oxidases (ACOXs) genes in *rsks-1(ok1255)* mutants (GSE272718) by heatmap analysis. Color coding reflects relative representation in RNA-seq data (Log_2_(Fold Change)), with red and blue indicating increased and decreased expression, respectively. Log_2_FC ≥ |0.75|, *p* < 0.05. (**D**) Representative images of *acox-1.2p::gfp*. Scale bars: 100 μm (applies to all panels). (**E**) Knockdown of *rsks-1* by RNAi resulted in increased expression of the *acox-1.2p::gfp* reporter. (**F**) Representative images of *prx-11p::prx-11::gfp*. Scale bars: 10 μm (applies to all panels). (**G**) Knockdown of *rsks-1* by RNAi resulted in increased the size of peroxisomes in worms expressing *prx-11p::prx-11::gfp*. (**H**) Knockdown of *rsks-1* by RNAi resulted in increased number of peroxisomes in worms expressing *prx-11p::prx-11::gfp*. Data were presented as mean values ± SD of three independent experiments (*n* = 30 worms per experiment). *** *p* < 0.001, ** *p* < 0.01. *p*-values were calculated using the two-sample *t*-test.

As demonstrated schematically, both the intracellular sources and detoxification pathways of H_2_O_2_ were identified as being pivotal to the metabolic balance of H_2_O_2_ (Figure 2A). The generation of H_2_O_2_ occurs across a variety of subcellular compartments, including the plasma membrane, mitochondria, endoplasmic reticulum (ER), and peroxisomes [15,36,37,38]. In addition, the subsequent detoxification of H_2_O_2_ is carried out by catalases (CTLs), glutathione peroxidases (GPXs), peroxiredoxins (PRDXs), and the thioredoxin (TRX) system [39,40]. By analyzing existing transcriptome data of long-lived mutants (GSE272718, GSE93724, GSE293218, and GSE63075), we observed that the oxidative stress response genes were upregulated in long-lived *rsks-1(ok1255)* and *glp-1(e2141ts)* mutants (Figure 2B and Figure S1). Importantly, the mRNA levels of peroxisomal acyl-CoA oxidase genes were upregulated in *rsks-1(ok1255)* and *glp-1(e2141ts)* mutants, rather than other long-lived mutants (Figure 2C and Figure S2). In addition, we analyzed the single-nucleus transcriptomic dataset (GSE229022). The results showed that peroxisomal acyl-CoA oxidase genes and oxidative stress response genes were upregulated in most tissues of the *rsks-1(ok1255)* mutant worms (Figure S3). To validate these findings, we employed transgenic *C. elegans* expressing *acox-1.2p::gfp*, in which *acox-1.2* represents a key gene involved in the peroxisomal fatty acid β-oxidation pathway, as well as *prx-11p::prx-11::gfp* transgenic worms, which serve as a reporter for peroxisome biogenesis. Upon *rsks-1* knockdown, the transcriptional expression of *acox-1.2* was significantly upregulated, accompanied by a marked increase in both the number and size of peroxisomes (Figure 2D–H).

**Figure 3:**
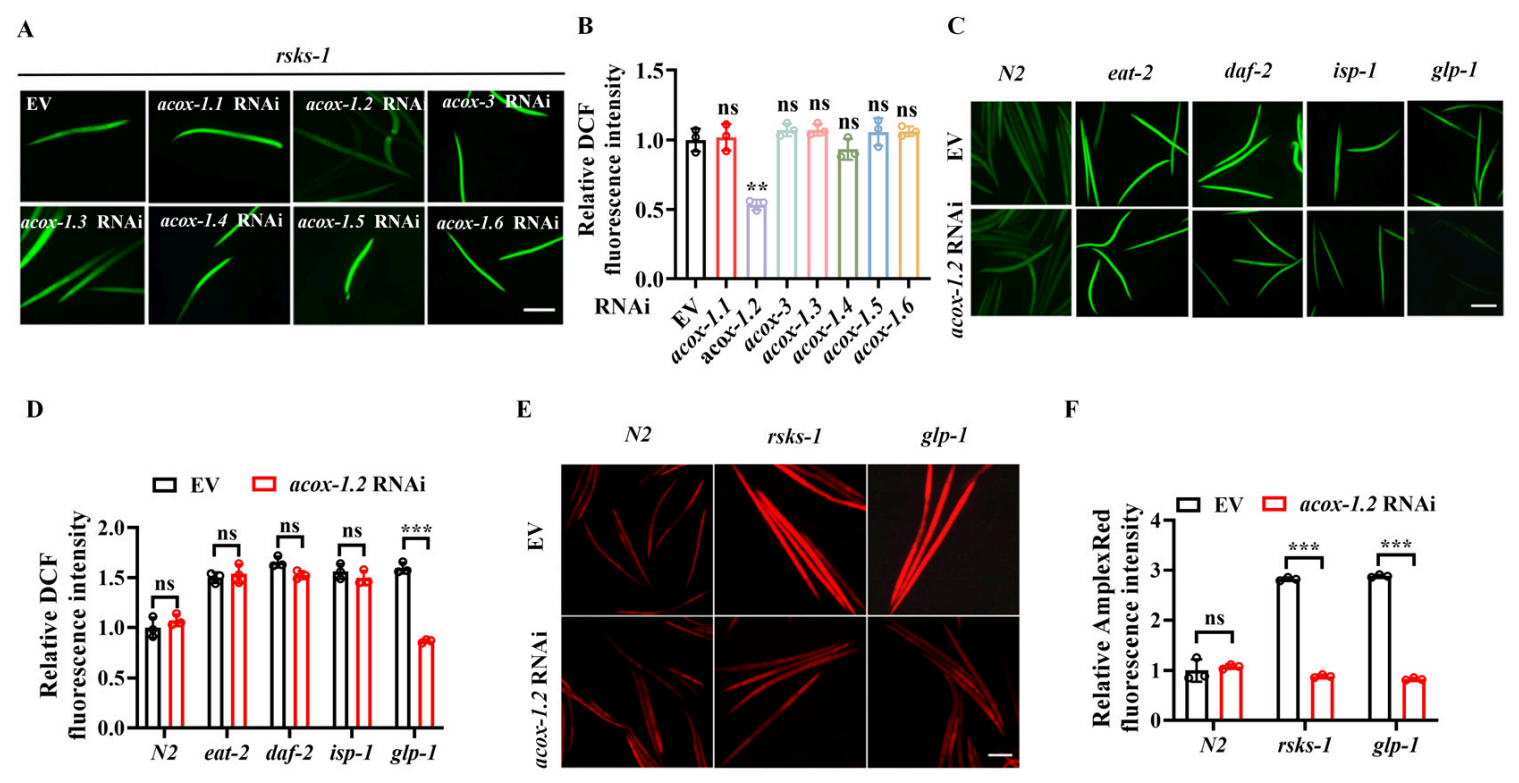
ACOX-1.2 is required for H_2_O_2_ production in long-lived *rsks-1(ok1255)* and *glp-1(e2141ts)* mutant worms (**A**) RNAi screening of the seven acox acyl-CoA oxidase genes (*acox-1.1*, *acox-1.2*, *acox-1.3*, *acox-1.4*, *acox-1.5*, *acox-1.6*, and *acox-3*) in *rsks-1 (ok1255)* mutants. Representative images of 2′,7′-dichlorofluorescein diacetate (H_2_DCFDA) staining in worms. Scale bars: 100 μm (applies to all panels). (**B**) Quantification of the fluorescence intensity of H_2_DCFDA. (**C**) Knockdown of *acox-1.2* by RNAi reduces the ROS levels in *rsks-1(ok1255)* and *glp-1(e2141ts)* mutants, but has no effect on the other long-lived mutants (*daf-2(e1370)*, *eat-2(ad1116)*, and *isp-1(qm150)*). Representative images of 2′,7′-dichlorofluorescein diacetate (H_2_DCFDA) staining in worms. Scale bars: 100 μm (applies to all panels). (**D**) Quantification of the fluorescence intensity of H_2_DCFDA. Data were presented as mean values ± SD of three independent experiments (*n* = 30 worms per experiment). (**E**) Knockdown of *acox-1.2* by RNAi reduces the H_2_O_2_ levels in long-lived *rsks-1(ok1255)* and *glp-1(e2141ts)* mutants. Representative images of 10-acetyl-3,7-dihydroxy-phenoxazine (Amplex Red) staining in worms. Scale bars: 100 μm (applies to all panels). (**F**) Quantification of the fluorescence intensity of Amplex Red. Data were presented as mean values ± SD of three independent experiments (*n* = 30 worms per experiment). ** *p* < 0.01, *** *p* < 0.001, ns, not significant. *p*-values are calculated using a one-way ANOVA followed by a Student’s *t* test.

To determine which ACOX contributes to H_2_O_2_ formation in *rsks-1(ok1255)* mutants, we knocked down the upregulated acox genes by RNAi, including *acox-1.1*, *acox-1.2*, *acox-1.3*, *acox-1.4*, *acox-1.5*, *acox-1.6*, and *acox-3*. Of these seven acox genes, *acox-1.2* RNAi, rather than the other six genes, significantly reduced the signal intensity of H_2_DCFDA in *rsks-1(ok1255)* mutants (Figure 3A,B). The signal intensity of H_2_DCFDA was reduced in *glp-1(e2141ts)* mutants by *acox-1.2* RNAi, but not in the other long-lived mutants and WT worms (Figure 3C,D). Moreover, Amplex Red revealed that the levels of H_2_O_2_ were significantly reduced in *rsks-1(ok1255)* mutants by *acox-1.2* RNAi (Figure 3E,F), suggesting that ACOX-1.2 is required for H_2_O_2_ production in *rsks-1(ok1255)* mutants. In addition, we validated the knockdown efficiency of all RNAi strains by RT–qPCR (Figure S4). The results indicate that the levels of H_2_O_2_ were elevated in *rsks-1(ok1255)* and *glp-1(e2141ts)* mutants, a phenomenon that appears to be contingent upon the enhanced mRNA levels of peroxisomal acyl-CoA oxidase ACOX-1.2. The metabolic balance of H_2_O_2_ in peroxisomes is disrupted in long-lived *rsks-1(ok1255)* and *glp-1(e2141ts)* mutants.

### 3.3. Acyl-CoA Oxidase ACOX-1.2 Is Imperative for the Elevated Autophagy in rsks-1 Mutants

**Figure 4:**
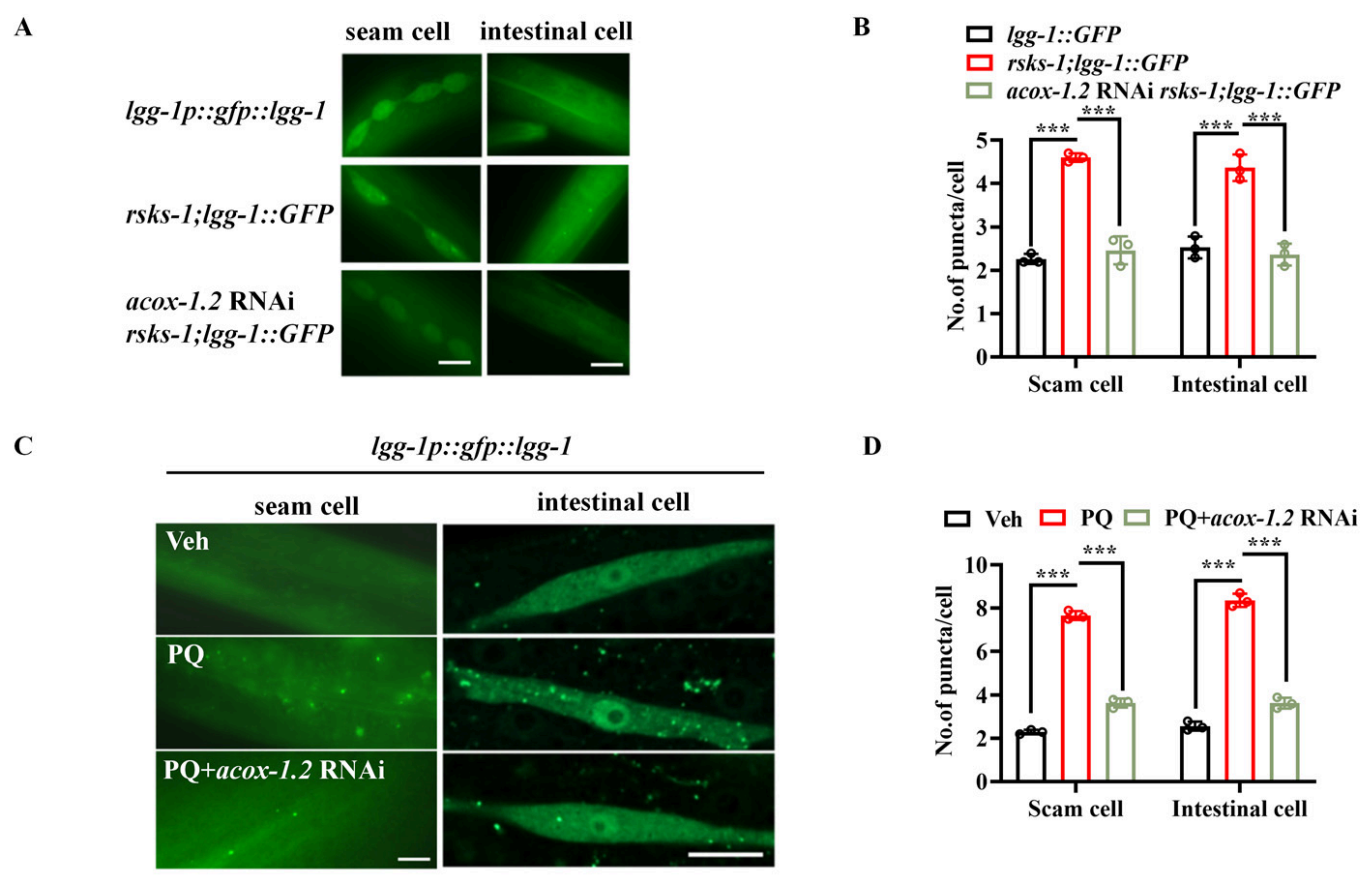
ACOX-1.2 is required for the elevated autophagy in *rsks-1(ok1255)* mutants. Knockdown of *acox-1.2* by RNAi suppresses the numbers of autophagy in *rsks-1(ok1255)* mutants and worms treated with 0.25 mM paraquat (PQ). (**A**) Representative images of autophagosomes (GFP::LGG-1 puncta) in the seam cells and intestinal cells of *rsks-1(ok1255)* mutants. Scale bars: 10 μm (applies to all panels). (**B**) Quantification of the number of GFP::LGG-1 puncta in the seam cells and intestinal cells of *rsks-1(ok1255)* mutants. (**C**) Representative images of autophagosomes (GFP::LGG-1 puncta) in the seam cells and intestinal cells of worms treated with PQ. Seam cell scale bars: 10 μm (applies to all panels). Intestinal cell scale bars: 10 μm (applies to all panels). (**D**) Quantification of the number of GFP::LGG-1 puncta in the seam cells and intestinal cells of worms treated with PQ. Data were presented as mean values ± SD of three independent experiments (*n* = 30 worms per experiment). *** *p* < 0.001. *p-*values were calculated using a one-way ANOVA followed by a Student’s *t* test.

Previous studies have shown that peroxisomes promote autophagy via H_2_O_2_ as a signaling molecule, thereby extending lifespan in worms [9]. Therefore, we used transgenic worms expressing GFP::LGG-1 to assess autophagy levels. As is well known, in the course of autophagy, LGG-1/ATG8 is sequestered at the membrane of the resulting autophagosomes, condensing into a series of puncta [41,42]. Quantification of the number of GFP puncta revealed significantly increased autophagosome accumulation in both seam cells and intestinal cells of *rsks-1(ok1255)* mutants compared with WT worms (Figure 4A,B). Knockdown of *acox-1.2* by RNAi markedly reduced the number of GFP puncta in both seam cells and intestinal cells of *rsks-1(ok1255)* mutants (Figure 4A,B), suggesting that ACOX-1.2 is required for the elevated autophagy in *rsks-1* mutants.

Next, we investigated the hypothesis that the presence of ROS not derived from peroxisomes would result in increased autophagy activity, which would in turn extend the lifespan of worms, but it was independent of the acyl-CoA oxidase ACOX-1.2. As expected, treatment with a life-extending dose (0.25 mM) of paraquat (an ROS generator) stimulated autophagy formation in both seam cells and intestinal cells of GFP::LGG-1 transgenic worms (Figure 4C,D) [18]. However, the knockdown of *acox-1.2* by RNAi led to the partial suppression of paraquat-induced autophagy (Figure 4C,D). The results indicate that paraquat may have the effect of activating acyl-CoA oxidase ACOX-1.2.

### 3.4. Acyl-CoA Oxidase ACOX-1.2 Is Essential for Extending the Lifespan of rsks-1 Mutants

**Figure 5:**
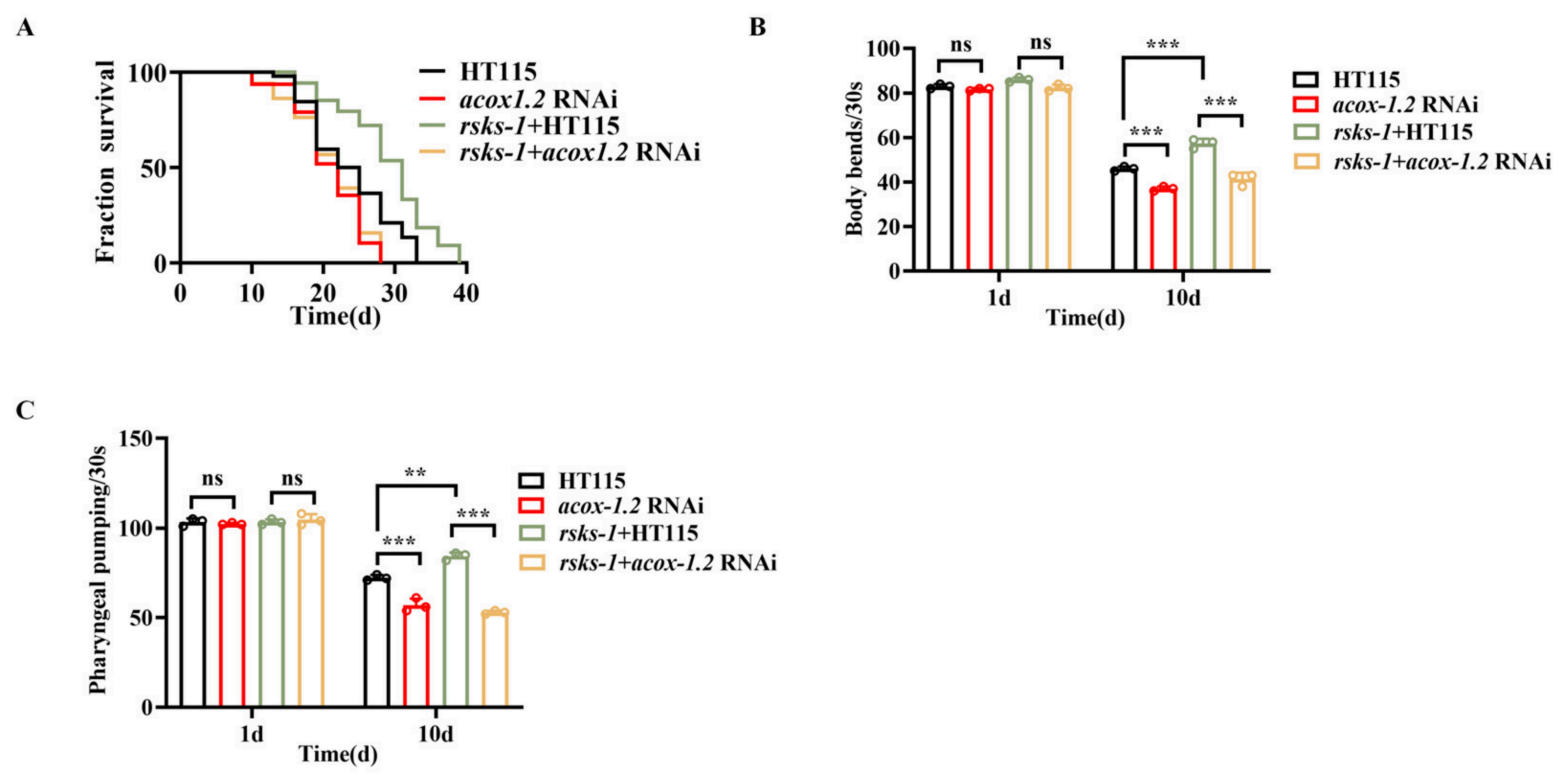
ACOX-1.2 is essential for extending the lifespan of *rsks-1(ok1255)* mutants. (**A**) *acox-1.2* RNAi suppresses the lifespan of *rsks-1(ok1255)* mutants and wild-type (WT) worms. *p* < 0.05 *acox-1.2* RNAi Vs empty vector (EV), *p* < 0.001 *rsks-1* + *acox-1.2* RNAi Vs *rsks-1* + EV. *p-*values were calculated using a log-rank test. (**B**) *acox-1.2* RNAi reduces the body bending frequency in 10-days- *rsks-1(ok1255)* mutants and WT worms. (**C**) *acox-1.2* RNAi reduces the pharyngeal pumping rate in 10-days- *rsks-1(ok1255)* mutants and WT worms. Data were presented as mean values ± SD of three independent experiments (*n* = 40–60 worms per experiment). ** *p* < 0.01, *** *p* < 0.001, ns, not significant. *p*-values are calculated using a one-way ANOVA followed by a Student’s *t* test.

We tested whether hydrogen peroxide generated during peroxisomal β-oxidation drives the extended lifespan seen in *rsks-1(ok1255)* mutant worms. Knocking down *acox-1.2* via RNAi shortened the lifespan of wild-type animals and fully blunted the longevity gain of *rsks-1(ok1255)* mutants (Figure 5A). Pharyngeal pumping and body bending are commonly used healthspan indicators in *C. elegans* that decline with age, and the rate of their decline is negatively correlated with lifespan [43,44]. We further assessed how ACOX-1.2 shapes age-related physiological traits—namely, pharyngeal pumping and body bending, as previously reported [45]. As anticipated, the age-dependent decline in both pharyngeal pumping (Figure 5B) and body bending (Figure 5C) was attenuated in 10-day-old *rsks-1(ok1255)* mutants—an effect abrogated by *acox-1.2* RNAi in both genotypes. Together, these data establish that peroxisomal β-oxidation-derived H_2_O_2_ is a critical determinant of the *rsks-1(ok1255)* longevity phenotype.

## 4. Discussion

The present study corroborates the findings of previous studies showing that ROS functions as a pivotal signaling molecule in the regulation of longevity in worms [9,14,18]. Most importantly, inhibition of peroxisomal fatty acid β-oxidation suppressed both autophagy and lifespan extension in the long-lived *rsks-1(ok1255)* and *glp-1(e2141ts)* mutant worms [9]. The findings reveal that the production of H_2_O_2_ by the peroxisomal enzyme ACOX-1.2 plays a crucial role in autophagy-mediated lifespan regulation in worms. Although our findings support an association between ACOX-1.2-dependent H_2_O_2_ metabolism, autophagy, and lifespan extension, whether autophagy is genetically required for the lifespan-extending effect of ACOX-1.2 remains to be determined. Notably, our study provides novel insights into the role of ROS in aging by demonstrating that the peroxisomal H_2_O_2_ metabolic balance mediates lifespan regulation in worms.

This study showed that H_2_O_2_ levels were elevated in all long-lived mutant worms, including those associated with IIS, the rapamycin (TOR) pathway, germ-cell loss, the electron transport chain (ETC), and dietary restriction (DR). The generation of H_2_O_2_ is observed in a range of subcellular compartments, including the plasma membrane, mitochondria, ER, and peroxisomes [15,36,37,38]. At the plasma membrane, NADPH oxidases (NOXs) catalyze the transfer of electrons from NADPH to O_2_, thereby producing O_2_^−^, which is subsequently dismutated to H_2_O_2_ [36,46,47]. Mitochondria represent another major source, where electron leakage from the ETC, especially complexes I and III, leads to O_2_^−^ generation that is rapidly dismutated into H_2_O_2_ by mitochondrial superoxide dismutase (SOD-2 and SOD-3) [15]. The ER serves as the central site for the synthesis and folding of secretory and membrane proteins. To ensure proper protein conformation, the formation of numerous disulfide bonds is required. During this process, the key catalysts-protein disulfide isomerase (PDI) and its partner ERO1-facilitate thiol oxidation to form disulfide bonds, transferring electrons ultimately to molecular oxygen and thereby inevitably generating H_2_O_2_ [37]. Peroxisomes generate H_2_O_2_ primarily through acyl-CoA oxidases involved in fatty acid β-oxidation, which transfer electrons directly to O_2_ [9]. The analysis of transcriptomic data from the long-lived mutant worms revealed that the mRNA levels of seven acyl-CoA oxidase genes (*acox-1.1*, *acox-1.2*, *acox-1.3*, *acox-1.4*, *acox-1.5*, *acox-1.6*, and *acox-3*) were elevated in long-lived *rsks-1(ok1255)* and *glp-1(e2141ts)* mutants. Moreover, we found that only RNAi-mediated knockdown of the acyl-CoA oxidase gene *acox-1.2* significantly reduced H_2_O_2_ levels in the long-lived *rsks-1(ok1255)* and *glp-1(e2141ts)* mutants. Notably, *acox-1.2* RNAi did not affect H_2_O_2_ levels in the other long-lived mutant strains. These results suggest that the increase in H_2_O_2_ observed in the *rsks-1(ok1255)* and *glp-1(e2141ts)* mutants is primarily attributable to peroxisomal fatty acid β-oxidation, whereas the elevated H_2_O_2_ levels in the other long-lived mutants are likely derived from the plasma membrane, mitochondria, or the endoplasmic reticulum (ER).

The “oxidative stress theory of aging” or “free radical theory of aging” is widely accepted, given that ROS have the potential to cause oxidative damage [19,48]. However, there is currently insufficient evidence to confirm that oxidative damage is the cause of aging. It has been hypothesized that oxidative stress is predominantly mediated by ROS generated in mitochondria [49]. Nevertheless, it is conceivable that oxidative stress may be initiated by an imbalance between ROS production and clearance [19]. The *C. elegans* genome contains three CTLs, four GLRXs, eight TRXs, and three PRDXs [19], which are involved in the detoxification of H_2_O_2_. For instance, the β-oxidation of fatty acids in peroxisomes results in the production of H_2_O_2_, while peroxisomal CTLs are responsible for the clearance of H_2_O_2_, thereby ensuring the maintenance of the metabolic balance of H_2_O_2_. In fact, the metabolic balance of H_2_O_2_ in peroxisomes is disrupted in long-lived *rsks-1(ok1255)* and *glp-1(e2141ts)* mutant worms. However, the imbalance of H_2_O_2_ in peroxisomes contributes to extending the lifespan of *C. elegans*, a phenomenon that does not align with the theories of the “oxidative stress theory of aging” or “free-radical theory of aging”.

Previous studies have shown that PKA activation inhibits AMPK-driven autophagy, thereby promoting *C. elegans* motility and longevity in the early stages of fasting [50]. The H_2_O_2_ promotes autophagy in worms by activating the ROS-sensitive transcription factor SKN-1/NRF2. SKN-1/NRF2 has been shown to play a significant role in promoting longevity and delaying senescence in both worms and mammals [51,52]. Moreover, SKN-1/NRF2 has been identified as one of the master regulators of autophagy in both worms and mammals [9,53]. To assess whether SKN-1 activation in *rsks-1*-deficient worms depends on peroxisomal fatty acid β-oxidation, using its downstream target gene *gst-4* as a transcriptional reporter. We found that *rsks-1* knockdown significantly increased the fluorescence intensity of *gst-4p::gfp*. Importantly, this fluorescence was markedly reduced in *rsks-1* RNAi worms subjected to *acox-1.2* RNAi (Figure S5). Our hypothesis is that the production of H_2_O_2_ by peroxisomal fatty acid β-oxidation serves as a key signaling molecule in the regulation of the SKN-1-autophagy axis-mediated lifespan in *rsks-1*-deficient worms.

## Data Availability

The datasets used and/or analyzed during the current study were available from the corresponding author on reasonable request.

## Supplementary Materials

The following supporting information can be downloaded at https://www.mdpi.com/article/10.3390/cimb48090909/s1.
