# Supplementary material for: The Extended Lifespan of Caenorhabditis elegans rsks-1 Mutants Depends on the Peroxisomal Fatty Acid β-Oxidation Enzyme ACOX-1.2
Source: Curr Issues Mol Biol. 2026 Sep 4;48(9):909. doi: 10.3390/cimb48090909 (PMC13605461; doi:10.3390/cimb48090909)
Supplement: Supplementary file 1 [file cimb-48-00909-s001.zip › Supplementary figures.pdf]

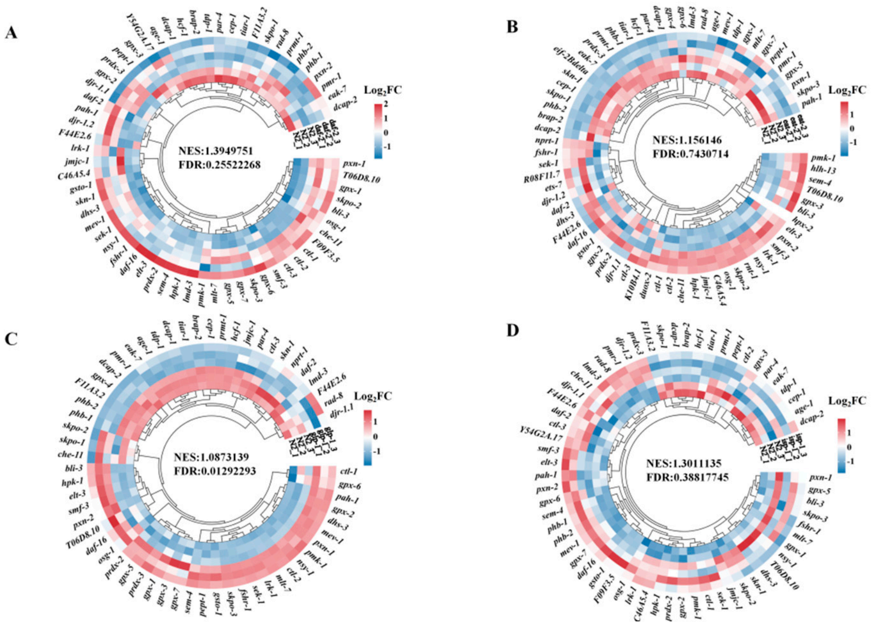

Supplementary Figure S1. Expression patterns of oxidative stress response genes in the long-lived mutant worms.

(A-D) The expression pattern of the oxidative stress response genes in *daf-2(e1370)* (A), *eat-2(ad1116)* (B), *glp-1(e2141)* (C), and *isp-1(qm150)* (D) mutants by GSEA. Color coding reflects relative representation in RNA-seq data ($Log_2^{(Fold\ Change)}$), with red and blue indicating increased and decreased expression, respectively. $Log_2FC \geq |0.75|$, $P < 0.05$.

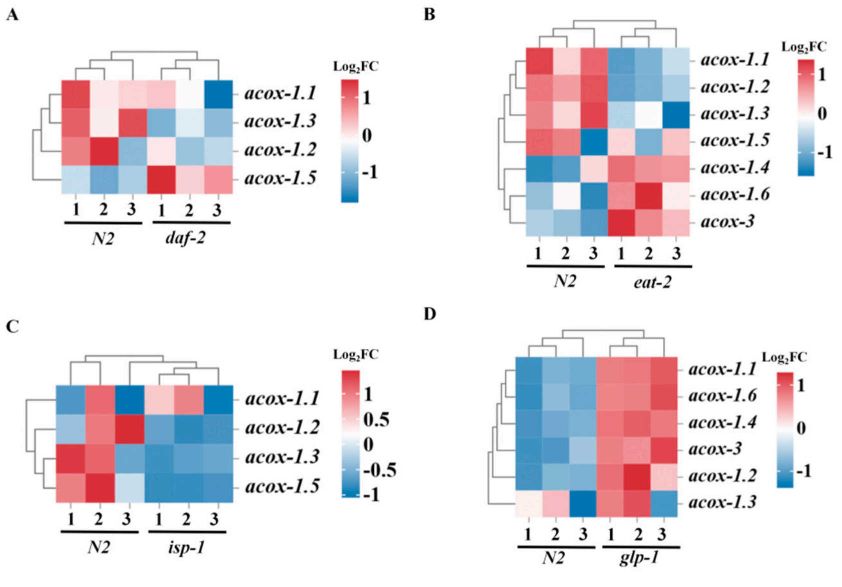

Supplementary Figure S2. Expression patterns of acyl-CoA oxidase genes in the

long-lived mutant worms.

(A-D) The expression pattern of the acyl-CoA oxidase genes in *daf-2(e1370)* (A), *eat-2(ad1116)* (B), *glp-1(e2141)* (C), and *isp-1(qm150)* (D) mutants by heatmap analysis. Color coding reflects relative representation in RNA-seq data ($Log_2^{(Fold\ Chang)}$), with red and blue indicating increased and decreased expression, respectively. $Log_2FC \geq |0.75|$, $P < 0.05$.

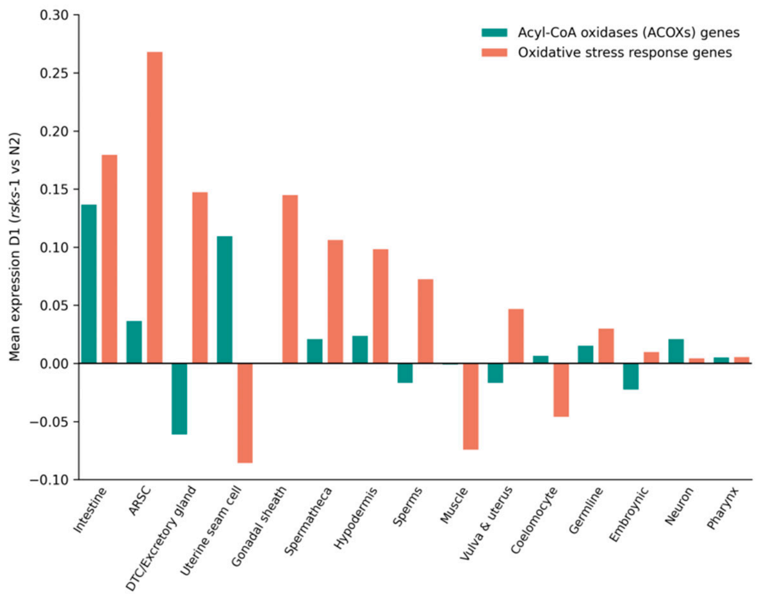

Supplementary Figure S3. Expression patterns of acyl-CoA oxidase genes and oxidative stress response genes in *rsks-1 (ok1255)* mutants.

The expression pattern of the acyl-CoA oxidase genes and oxidative stress response genes in *rsks-1 (ok1255)* mutant worms by mean expression analysis. The length of each bar represents the mean expression level of the indicated gene category in single cells from the corresponding tissue. Different colors indicate different pathway-related gene groups. Green represents the peroxisomal acyl-CoA oxidase genes, whereas red represents the oxidative stress response genes.

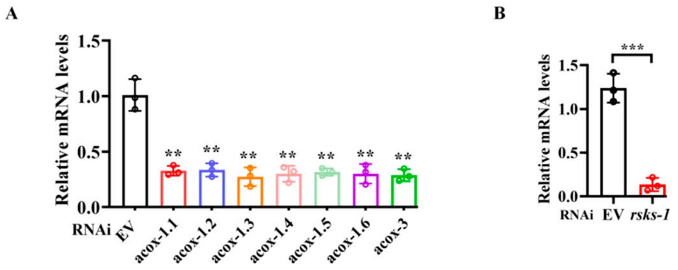

Supplementary Figure S4. The knockdown efficiency was validated by RT-qPCR.

(A) Validation of the RNAi knockdown efficiency of the ACOX family genes. (B) Validation of the RNAi knockdown efficiency of rsks-1.Data are presented as mean values±SD of three independent experiments. **P < 0.01, ***P < 0.001. P-values throughout were calculated using the two-sample t-test.

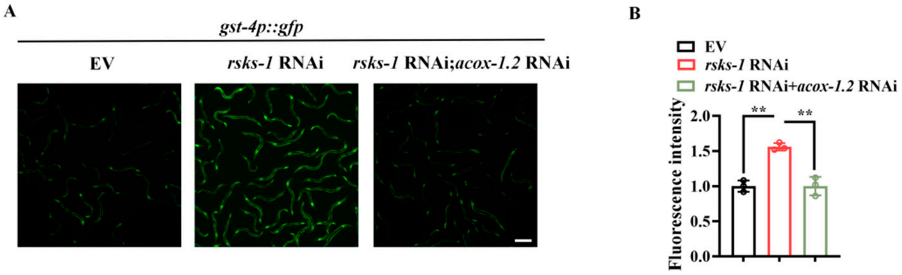

Supplementary Figure S5. The expression of *gst-4p::gfp* was markedly reduced in *rsks-1* RNAi worms subjected to *acox-1.2* RNAi.

(A) Representative images of *gst-4p::gfp* fluorescence. (B) Quantification of GFP fluorescence intensity. Data are presented as mean ± SD of three independent experiments (n=30 worms per experiment). ***P* < 0.01. *P*-values were calculated using one-way ANOVA followed by an unpaired Student's t-test.
